# Therapeutic Values of Earthworm Species Extract from Azad Kashmir as Anticoagulant, Antibacterial, and Antioxidant Agents

**DOI:** 10.1155/2022/6949117

**Published:** 2022-02-07

**Authors:** Rozina Ghulam Mustafa, Andleeb Dr Saiqa, Jorge Domínguez, Madiha Jamil, Saira Manzoor, Samna Wazir, Bushra Shaheen, Asma Parveen, Rida Khan, Shaukat Ali, Nazish Mazhar Ali, Fatima Jalal, Sadaf Azad Raja

**Affiliations:** ^1^Microbial Biotechnology and Vermi-Technology Laboratory, Department of Zoology, University of Azad Jammu and Kashmir, Muzaffarabad 13100, Pakistan; ^2^Grupo de Ecoloxía Animal (GEA), Universidade de Vigo, Vigo E-36310, Spain; ^3^Department of Zoology, Government College University, Lahore, Pakistan; ^4^Department of Zoology, Government College University, Faisalabad, Pakistan; ^5^Department of Biosciences, COMSATS University, Islamabad, Pakistan

## Abstract

**Aims:**

Current research aimed to explore the therapeutic values of different earthworms as antibacterial, anticoagulant, and antioxidant agents.

**Methods:**

Ten different earthworms, i.e., *Amynthas corticis, Amynthas gracilis, Pheretima posthuma, Eisenia fetida, Aporrectodea rosea, Allolobophora chlorotica, Aporrectodea trapezoides, Polypheretima elongata, Aporrectodea caliginosa*, and *Pheretima hawayana*, were collected and screened for biological activities. Antibacterial effect analysis of earthworm species was done against fourteen bacterial pathogens, i.e., *Escherichia coli, Serratia marcescens, Streptococcus pyogenes, Staphylococcus epidermidis, Staphylococcus aureus, Klebsiella pneumoniae, Pseudomonas aeruginosa (1), Salmonella typhimurium, Shigella flexneri, Enterobacter amnigenus, Serratia odorifera, Pseudomonas aeruginosa (2), Staphylococcus warneri*, and *Lactobacillus curvatus*, *via* agar well diffusion, crystal violet, MTT, agar disc diffusion, and direct bioautography assays. Antioxidant potential was evaluated through ABTS and DPPH assays. Lipolytic, proteolytic, and amylolytic assays were done for lipase, protease, and amylase enzymes confirmation. *In vitro* anticoagulant effects were examined in the blood samples by measuring prothrombin time.

**Results:**

Results revealed that all earthworm extracts showed the inhibition of all tested bacterial pathogens except *P. aeruginosa (1), P. aeruginosa (2), S. warneri,* and *L. curvatus*. The maximum zone of inhibition of *E. coli* was recorded as 14.66 ± 0.57 mm by *A. corticis*, 25.0 ± 0.0 mm by *P. posthuma*, 20.0 ± 0.0 mm by *E. fetida*, and 20.0 ± 0.0 mm by *A. trapezoid.* Cell proliferation, biofilm inhibition, the synergistic effect of extracts along with antibiotics, and direct bioautography supported the results of agar well diffusion assay. Similarly, *P. hawayana, A. corticis*, *A. caliginosa,* and *A. trapezoids* increase the prothrombin time more efficiently compared to other earthworms. *A. corticis, A. gracilis, A. rosea, A. chlorotica, P. elongata,* and *A. trapezoides* showed maximum DPPH scavenging potential effect.

**Conclusions:**

The coelomic fluid of earthworms possessed several bioactive compounds/enzymes/antioxidants that play an important role in the bacterial inhibition and act as anticoagulant agents. Therefore, the development of new therapeutic drugs from invertebrates could be effective and potential for the prevention of the emergence of multidrug-resistant bacteria.

## 1. Introduction

Infection is caused by disease-causing agents such as bacteria, fungi, parasites, or viruses which are called infectious agents [1–3]. In many circumstances, infectious diseases can be transferred from person to person, either directly *via* skin contact or indirectly *via* contaminated water or food [4], and being exposed to organisms [5, 6]. Food-borne bacterial diseases are caused by *S. aureus, E. coli, Salmonella* species*, K. pneumonia, Listeria monocytogenes,* and *Campylobacter* species [7–10]. The recent antimicrobial research findings verified that bacteria can be the cause of nosocomial as well as community-acquired infections and have become a clinical threat to humans [11–13].

**Figure 1:**
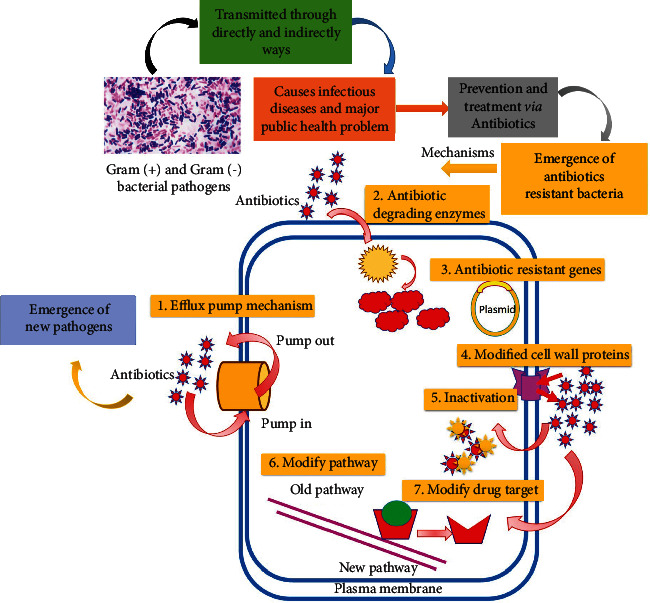
The emergence of new and antibiotic-resistant bacterial pathogens.

**Figure 2:**
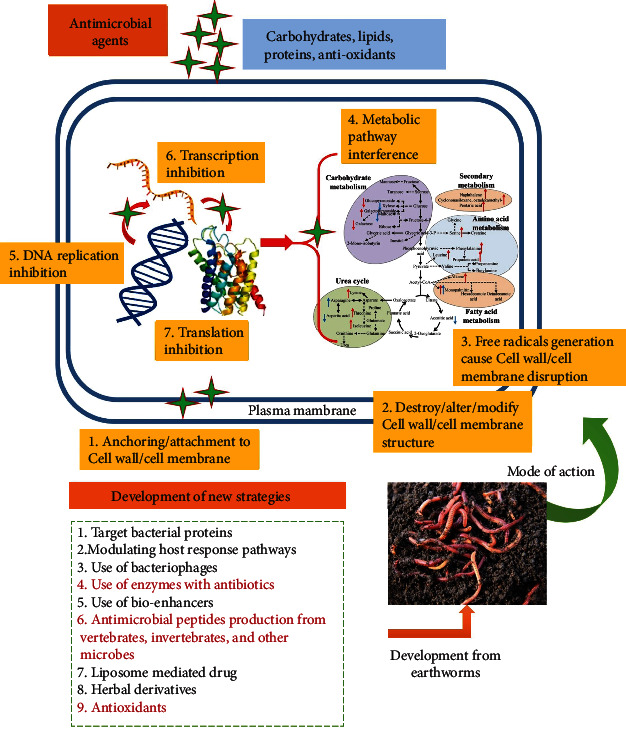
Prevention of bacterial transmission and its treatments *via* developing new strategies using earthworm species.

Antibiotics are one of the most important weapons fighting against bacterial infections [14] and have great benefits for the health. The effectiveness of antibiotics is threatened due to the rapid development of resistant bacteria worldwide [15, 16]. The antibiotic resistance has been attributed to the misuse of the medications, as well as a shortage of new drug development by the pharmacological industry [17–19]. As we observed, most antibiotics were discovered by using traditional methods which not only led to the emergence of drug resistance problem but also are involved in the emergence of new pathogens, i.e., multidrug-resistant bacteria. The mechanism can be categorized as (1) modification of drug target site [20, 21], (2) inactivation of antibiotics by enzymatic modifications [22–24], (3) decreased penetration of antibiotics because of cell wall proteins alteration [25, 26], (4) the presence of antibiotic-resistant genes carriers (plasmids) [27, 28], (5) activating the efflux pump mechanism [29], (6) modification in metabolic pathways [30], and (7) the presence of antibiotic degrading enzymes [31, 32] (Figure 1). So, researchers are trying to develop new strategies (Figure 2) for the antibacterial drug products based on new targets such as bacterial proteins, modulating host response pathways, using bacteriophages to treat bacterial infections [33], use of enzymes with antibiotics [34], use of bioenhancers [35, 36], antimicrobial peptides production from vertebrates, invertebrates, and other microbes [37], hybrid antibacterial drug [38], liposome-mediated drug [39], and herbal derivatives [40–43]. Similarly, antioxidants, i.e., flavonoids, tannins, anthocyanins, and phenolic acids, have also been reported as antioxidant, antibacterial, anti-inflammatory, antiviral, and anticancer agents [43] and have gained special attention over the last decades [44]. Phenolic compounds derived from lignocellulosic waste have been reported as an antioxidant and antimicrobial agent against food-borne pathogens [45]. Cooper et al. [46] illustrated the presence of antimicrobial and anticancer molecules in the earthworms. Similarly, several species of earthworms were screened for antimicrobial activities by Kathireswari et al. [47], Istiqumah et al. [48], Verma and Verma [49], and Chauhan et al. [50].

Although numerous therapeutic drugs have been developed and approved by Food and Drug Administration as antimicrobial, antioxidant, and anticoagulant agents, these drugs have certain drawbacks, i.e., side effects, being expensive, and becoming a major health problem. Therefore, there is a need for the production of new antibacterial, antioxidant, and anticoagulative agents from natural resources like invertebrate that could be used against both infectious and noninfectious diseases. Therefore, the current study aimed to evaluate the biological activities of earthworms such as antibacterial, antioxidant, and anticoagulant activities because infectious and noninfectious illnesses are a major cause of mortality and morbidity worldwide.

## 2. Materials and Method

### 2.1. Chemicals, Reagents, and Equipment

All chemicals and reagents were obtained from Sigma Aldrich (Germany), Merck (Germany), Riedel-DeHaan company, and Sigma Aldrich (Switzerland). Ethanol (RDH), DPPH, MTT, and Muller Hinton agar media were used. Screwed cap reagent bottles, preservative jars, flasks, test tubes, test tube holders, and Petri dishes were used. 37°C shaker (Irmeco GmbH, Germany), 37°C incubator (MMM group Medcenter Enrich tungsten GmbH), analytical balance (SARTORIUS GMBM GOTTINGEN, Germany), laminar flow (ESCO Prod Model; EQU/03-EHC; Serial # 2000-0052), camera lucida, digital weighing machine (Jeweler Precision Balance Model: DH-V600A), steam sterilizer (autoclave), silica gel plates, soil pH meter, orbital shaker, and EDTA tubes (Atlas-Labovac; evacuated blood) were used.

### 2.2. Ethical Statement

All experiments have been designed to avoid distress, unnecessary pain, and suffering to the experimental animals. All procedures were conducted following international regulations referred to as Wet op de dierproeven (Article 9) of Dutch Law. The current study is approved by the ethical committee of Office of Research Innovation and Commercialization (ORIC), the University of Azad Jammu and Kashmir, vide no. 09/ORIC/2022, dated 13-1-2022.

### 2.3. Collection and Identification of Earthworms

Mature individuals belonging to ten earthworm species were collected from the soil by hand sorting method [51], preserved in absolute ethanol, and transported to the laboratories of Zoology Department, Government College University Faisalabad, Faisalabad, Pakistan, and Grupo de Ecoloxía Animal (GEA) at the Universidade de Vigo, Spain, for identification.

### 2.4. Preparation of Powder and Extract

The collected earthworm species were washed with running tap water, placed on wet filter paper for 24 h to remove the soil aggregates from their guts, and later dried in an incubator for 48 h at 55°C according to Andleeb et al. [52]. After incubation, earthworms were crushed into a fine powder. This powder was macerated in methanol for one week. The homogenized mixture was filtered using Whatman filter No. 1, and the filtrated sample was concentrated by incubating at 60°C. The dried crude extract (1 mg) was weighed and dissolved in 1 ml of dimethyl sulfoxide (DMSO) and used for further biological activities. Various concentrations such as 0.1 mg/ml, 0.5 mg/ml, and 1.0 mg/ml of earthworm extracts were used for minimum inhibitory concentration estimation through agar well diffusion method.

### 2.5. Antibacterial Assays

Fourteen bacterial pathogens such as *E. coli* (ATCC-25922)*, S. marcescens* (wild-type)*, Klebsiella pneumonia* (ATCC-1705), *P. aeruginosa* (1:ATCC-15442), *S. typhimurium* (ATCC 1331)*, S. flexneri* (spoiled fish product)*, E. amnigenus* (spoiled fish product)*, S. odor* (spoiled fish *product*)*, P. aeruginosa* (2: spoiled chick product), *S. warneri* (meat product), and *L. curvatus* (meat product) were collected from the Biotechnology Laboratory, University of Azad Jammu and Kashmir, Muzaffarabad, and used to evaluate the bactericidal effect of earthworm extracts. These pathological strains were isolated from human infectious samples (urine, pus, and blood) and spoilage food, i.e., fish, chicken, and red meat [53, 54].

#### 2.5.1. Agar Well Diffusion Test

The antibacterial effect of earthworm's extract was assayed by agar well diffusion method against bacterial pathogens [55]. For bacterial growth nutrient, agar (oxide: CMOO3) and nutrient broth media (Oxide: CM1) were used. The microbes were added to a nutrient broth medium where they grow and incubated for 24 h on a rotary shaker. The temperature of the rotatory shaker was 37°C. Then, this culture was mixed in a newly formed nutrient agar medium (NAM) at 45°C. The culture was dropped into purified Petri dishes, and all dishes were placed in laminar flow at room temperature for solidification. Three wells by the diameter of 5 mm in each plate were made by using a sterilized micropipette tip of 1 ml. In each prepared well, about 30 *µ*l of extract was put and then placed for 24 h at 37°C. According to Seeley et al. [56], the growth of bacteria was determined in 24–48 h, and the diameter of the inhibition zone in mm was also measured with the help of a ruler [57].

#### 2.5.2. Agar Disc Diffusion Method

Sensitivity test/antibiogram analysis against seven bacterial strains was evaluated by agar disc diffusion [58, 59] and antibiotics were used as a positive control. Different sets of antibiotics like aminoglycosides (gentamycin), Penicillin (amoxicillin), Ciprofloxacin (Fluoroquinolone), and Sulfamethoxazole were studied for antibacterial effect. Nutrient agar and nutrient broth media (oxide: CMOO3; oxide: CM1) were used for the growth of bacteria. These microbes are grown in a nutrient broth medium and incubated for 24 h at 37°C in a rotary shaker. Then, the culture was mixed in freshly prepared nutrient agar medium at 45°C and poured into sterilized Petri dishes. All dishes were placed at room temperature for solidification in laminar flow. Triplicates of each antibiotic were placed on agar plates and placed at 37°C overnight. The growth of microbes had been determined after 24 h and the diameter of the clear zone in millimeters was measured [56]. Hammer et al. [57] reported that the diameter of inhibition zones was measured with the help of scale.

#### 2.5.3. Synergistic Effect

The synergistic effect was similarly evaluated by the agar disc diffusion method with slight modifications [58]. Antibiotic discs were impregnated with 10 *µ*l extract and dried for few minutes. After the drying process, discs were put on solidified agar plates and sited for 24–48 h at 37°C. Microbial progress and diameter of clear zones had been measured with scale [56, 57].

#### 2.5.4. Crystal Violet Test

For biofilm inhibition assay, crystal violet test was used [60]. Tested pathogens were grown in nutrient broth medium (2 ml) overnight at 37°C. Chloramphenicol and nutrient broth were used for positive and negative controls, respectively. After incubation, the broth medium was detached and the attached cells were stained by adding 0.1% crystal violet (125 *µ*l), incubated for 10–15 min at room temperature, and washed with water to eradicate dye and extra separated cells. The crystal violet was mixed with 30% acetic acid after staining and then kept for 10-15 min at room temperature. 30% acetic acid had been used as blank in water. The absorbance of mixed crystal violet was counted at 550 nm by using a spectrophotometer.

#### 2.5.5. Cell Viability Assay

According to Gerlier and Thomasset [61], for evaluation of bacterial cell viability, tetrazolium salt (MTT) assay was used. For this purpose, MTT (0.2 mg/ml) was dissolved in DMSO and then incubated for 1–4 h at room temperature. The microbes (100 *µ*l) were grown up in a nutrient broth medium (3 ml) at 37°C for the whole night. After this, overnight bacterial growth (100 *µ*l) was grown in freshly prepared nutrient broth medium (1 ml) and incubated at 37°C in a shaker for 3 h (exponentially growing cultures) at 150 rpm, and then 100 *µ*l incubated samples were added to every test sample. For initiation of the decreasing reaction, 10 *µ*l MTT solution was added and incubated for 2–4 h at 37°C (without shaking). During incubation, a purple color has shown which specified the creation of formazan crystals at room temperature. Later, 500 *µ*l of DMSO was added for the mixing of crystals. At the end at 570 nm using a spectrophotometer, the absorbance of every sample was measured. DMSO was used as a control.

### 2.6. Antioxidant Assays

#### 2.6.1. DPPH Assay

Free radical scavenging activity was measured using DPPH assay [62] with near modification. Three ml of DPPH (0.4 mg%) in methanol solution was added to 0.1 ml of the earthworm extract, mixed to homogenize, and placed in the dark for 20 min and absorbance was calculated at 517 nm by using a spectrophotometer (Ai). DPPH solution was also used as standard (Ao). The percentage scavenging activity was designed by the formula: percent = [(Ao − Ai)/Ao] × 100.

#### 2.6.2. ABTS Assay

ABTS^**+**^ scavenging action was analyzed to calculate the antioxidant potential of earthworm extracts, according to the method of Re et al. [63]. The ABTS^**+**^ stock solution was made by reacting potassium persulphate and ABTS^**+**^. For the formation of ABTS^**+**^ free radicals, this mixture was allowed to stand for at least 16 hr. Then the running mixture was organized by diluting the stock solution with solvent methanol and the absorbance of this standard solvent was recorded at 734 nm (A0_Control_). For tests (A), 1 ml of ABTS^**+**^ running solution was homogenized with 10 *µ*l extracts and their absorbance was recorded at 734 nm. For blank (B), 10 *µ*l of the extract was mixed with distilled water and its absorbance was also observed at 734 nm. Test sample (Ai) for all extracts was calculated by subtracting the value of blank B from A. The percentage radical scavenging activity (% RSC) was measured using the formula: % RSC = [(A0_Control_ − Ai_Sample_)]/A0_Control_ × 100%.

### 2.7. Direct Bioautography

The agar overlay technique was used for the measurement of direct bioautography with slight modifications as described by Roopalatha and Vijay-mala [64]. The TLC-developed plates having a separation of chemicals through the use of the abovementioned five solvent systems were kept in sterilized Petri dishes. Then, overnight culture (*S. epidermidis, E. coli,* and *K. pneumonia*) was freshly prepared and was homogenized with nutrient agar and decanted over a chromatogram as a thin layer. These plates were kept at room temperature for 5 min and then incubated overnight at 37°C. The growth inhibition zones were measured around the active chromatogram spots. The antibacterial activity of constituents present in the spot was further confirmed by spraying TBTB solution (Thiazolyl blue tetrazolium Bromide) on Petri plates and these plates were incubated at 37°C for 4 h.

### 2.8. Anticoagulant Assay

*In vitro* anticoagulant effect of earthworm extracts were observed in the blood samples by evaluating prothrombin time [65]. About 10 ml of blood was drawn from healthy volunteers by making vein puncture using sterile syringes. Blood was collected in a PT tube containing 3.8% trisodium citrate solution to avoid the natural coagulation process. Immediate centrifugation was carried out for 15 min at a rate of 3000 rpm. After centrifugation, blood cells were discarded and plasma was collected. Plasma was used for PT examination. The sample of plasma was separated into two groups: Group I: negative control, Group II: earthworm extracts. A water bath was used for incubating the tubes with a mixture at 37°C. To analyze the clot for every 30 sec all the tubes were tilted at an angle of 45°. Clot formation time was measured by using a stopwatch. This time is called PT. Tests were repeated 3 times and the average time was calculated.

### 2.9. Statistical Analysis

Each experiment was repeated in triplicate. Mean ± standard deviation from absolute data was calculated using an online calculator (http://easycalculation.com/statistics/standard-deviation.php). The statistical significance was evaluated by one-way analysis of variance (ANOVA) at *p* ≤ 0.001 and MS Excel program was also used to plot graphs with error bars of standard errors of the means (SEM). For sensitivity tests, (0) was used for no sensitivity, ^*∗*^(>1–5 mm) for low sensitivity, ^*∗∗*^(>5–10 mm) for moderate sensitivity, and ^*∗∗∗*^(>10–25 mm) for high sensitivity.

## 3. Results

### 3.1. Identification of Earthworms

*Amynthas corticis, Amynthas gracilis, Pheretima hawayana. Pheretima posthuma*, and *Polypheretima elongata* (Megascolecidae) and *Eisenia fetida, Allolobophora chlorotica, Aporrectodea rosea, Aporrectodea trapezoides,* and *Aporrectodea caliginosa* (Lumbricidae) were identified by Prof. Dr. Jorge Domínguez (Spain) and Dr. Fatima Jalal (Pakistan) and further used for screening biological activities of their extracts.

### 3.2. Antibacterial Efficacy of Earthworms

**Figure 3:**
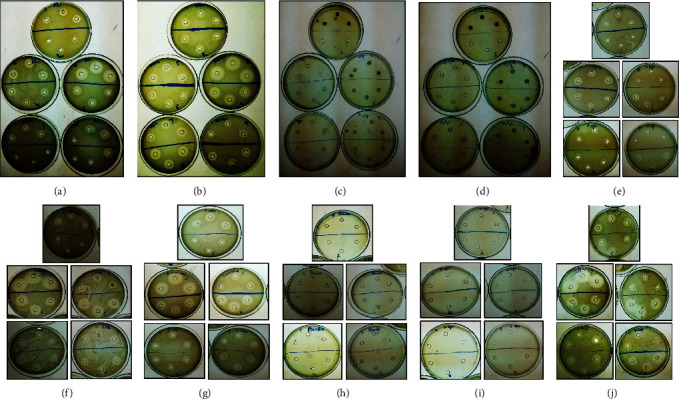
The bactericidal effect of earthworm species against pathogenic bacteria *via* agar well diffusion assay. (1) *Amynthas minimus*, (2) *Amynthas gracilis*, (3) *Pheretima posthuma,* (4) *Eisenia fetida,* (5) *Aporrectodea rosea*, (6) *Allolobophora chlorotica*, (7) *Aporrectodea trapezoides*, (8) *Pheretima lignicola*, (9) *Aporrectodea caliginosa,* and (10) *Pheretima hawayana.* (a) (b) *Shigella flexneri,* (c) *Pseudomonas aeruginosa,* (d) *Staphylococcus warneri*, (e) *Streptococcus pyogenes,* (f) *Klebsiella pneumoniae,* (g) *Serratia marcescens,* (h) *Pseudomonas aeruginosa*, (i) *Staphylococcus epidermidis*, and (j) *Escherichia coli*.

The bactericidal effect of ten earthworm species (*A. corticis, A. gracilis, P. posthuma, E. fetida, A. rosea, A. chlorotica, A. trapezoides, P. elongata, A. caliginosa*, and *P. hawayana*) was analyzed against fourteen bacterial pathogens such as *E. coli, S. marcescens, S. pyogenes, S. epidermidis, S. aureus, K. pneumoniae, P. aeruginosa (1), S. typhimurium, S. flexneri, E. amnigenus, S. odorifera, P. aeruginosa (2), S. warneri*, and *L. curvatus* through agar well diffusion method, biofilm inhibition, and cell proliferation inhibition assays. MICs results revealed that bactericidal effect of earthworm extracts was increased with increase concentration (Table 1). It was observed that all earthworm extracts had no antibacterial effect against *P. aeruginosa, S. warneri, L. curvatus,* and *S. epidermidis* at both 0.1 mg/ml and 0.5 ml/mg concentrations while low sensitivity was recorded at 1 mg/ml concentration used. On the other hand, all earthworm species showed antibacterial efficacy at 1 mg/ml used concentration and the zone of inhibition was recorded in the range of 2.0 mm - 25.0 mm around the wells (Table 1, Figure 3).

*Amynthas corticis* showed the maximum inhibition of *E. coli, S. marcescens*, and *E. amnigenus* with 14.66 ± 0.57 mm, 11.66 ± 0.57 mm, and 12.66 ± 0.57 mm zone of inhibition. On the other hand, moderate inhibition was recorded against *S. aureus* (6.0 ± 0.57 mm*), S. pyogenes* (7.0 ± 0.0 mm)*, S. odorifera* (9.0 ± 0.0 mm)*, K. pneumoniae* (7.66 ± 0.57 mm), and *S. typhimurium* (6.33 ± 1.15 mm). *Amynthas gracilis* showed moderate inhibition of *E. coli* (9.0 ± 1.0 mm), *S. odorifera* (7.0 ± 1.73 mm), and *K. pneumoniae* (5.66 ± 0.57 mm) (Table 1). *Pheretima posthuma* extract indicated the maximum growth inhibition of *E. coli, S. marcescens*, *K. pneumoniae*, *S. aureus*, *S. pyogenes*, *S. odorifera*, and *S. flexneri* as 25.0 ± 0.0 mm, 22.0 ± 0.0 mm, 25.0 ± 0.0 mm, 15.0 ± 0.0 mm, 15.0 ± 0.0 mm, 25.0 ± 0.0 mm, and 20.0 ± 0.57 mm, respectively (Table 1, Figure 3).

Similarly, *E. fetida* extract showed the maximum inhibition of *E. coli*, *S. marcescens*, *K. pneumoniae*, *S. aureus, E. amnigenus*, *S. odorifera,* and *S. flexneri* (20.0 ± 0.0 mm, 13.0 ± 0.0 mm, 10.33 ± 0.57 mm, 20.0 ± 0.0 mm, 15.0 ± 0.0 mm, 13.0 ± 0.57 mm, and 11.5 ± 0.57 mm), respectively, while moderate inhibition of *S. pyogenes* (8.0 ± 0.0 mm) and *S. typhimurium* (6.0 ± 0.0 mm) was recorded. *Aporrectodea rosea e*xtract showed maximum inhibition of *E. coli, S. marcescens, K. pneumoniae, S. pyogenes,* and *S. aureus* as 18.0 ± 0.0 mm, 16.0 ± 0.0 mm, 19.0 ± 0.0 mm, 13.0 ± 0.0 mm, and 15.0 ± 0.0 mm. On the other hand, a moderate zone of inhibition was recorded in the case of *P. aeruginosa* and *S. epidermidis* with 10.0 ± 0.0 mm and 10.0 ± 0.0 mm zone of inhibition.

*Aporrectodea trapezoides* showed the maximum inhibition of *E. amnigenus* (11.0 ± 0.0 mm). Bactericidal effect of *Allolobophora chlorotica* indicated the maximum inhibition of *E. coli, S. marcescens*, *S. aureus, E. amnigenus,* and *S. odorifera* (20.0 ± 0.0 mm, 16.0 ± 0.57 mm, 16.0 ± 0.57 mm, 25.0 ± 0.0 mm, and 11.0 ± 0.57 mm) while moderate sensitivity was measured against *K. pneumoniae* (10.0 ± 0.0 mm), *S. pyogenes* (8.66 ± 0.57 mm), *S. typhimurium* (6.0 ± 0.0 mm), and *S. flexneri* (6.0 ± 0.0 mm) (Table 1, Figure 3).

The extract of *P. elongata* showed the maximum growth inhibition of *K. pneumoniae*, *E. amnigenus,* and *S. flexneri* as 10.33 ± 0.57 mm, 13.33 ± 0.57 mm, and 10.33 ± 0.57 mm while moderate inhibition of *E. coli*, *S. marcescens, S. pyogenes*, and *S. aureus* were recorded (8.33 ± 0.57 mm, 6.33 ± 0.57 mm, 6.33 ± 0.57 mm, and 6.33 ± 0.0 mm)*. Aporrectodea caliginosa* extract showed the moderate zones of growth inhibition against *E. coli*, *S. marcescens*, *K. pneumoniae*, *S. typhimurium, S. flexneri,* and *E. amnigenus* (9.33 ± 0.57 mm, 5.66 ± 0.57 mm, 9.66 ± 0.57 mm, 9.33 ± 0.57 mm, 6.33 ± 0.57 mm, and 7.66 ± 0.57 mm, resp.) (Table 1). Similarly, *P. hawayana* extract showed the moderate inhibition of *E. coli*, *S. marcescens*, *K. pneumoniae*, *S. pyogenes*, *S. aureus, E. amnigenus, S. typhimurium, S. flexneri,* and *Serratia odorifera* (9.66 ± 0.57 mm, 7.33 ± 0.57 mm, 8.33 ± 0.57 mm, 7.33 ± 0.57 mm, 7.33 ± 0.57 mm, 9.66 ± 0.57 mm, 10.0 ± 0.0 mm, 10.0 ± 0.0 mm, and 7.66 ± 0.57 mm). On the other hand, it was observed that all earthworm extracts showed the lowest growth of inhibition of *P. aeruginosa (1), P. aeruginosa (2), S. warneri,* and *L. curvatus* (Table 1, Figure 3).

### 3.3. Antibiogram Analysis

Ciprofloxacin showed the maximum inhibition of *P. aeruginosa, K. pneumoniae, S. pyogenes, S. epidermidis,* and *S. aureus* (21.0 ± 0.0 mm, 17.0 ± 0.0 mm, 26.0 ± 0.0 mm, 19.0 ± 0.0 mm, and 27.0 ± 0.0 mm). Likewise, gentamycin indicated the maximum inhibition of *P. aeruginosa, K. pneumoniae, S. pyogenes, S. epidermidis,* and *S. aureus* (15.0 ± 0.0 mm, 17.0 ± 0.0 mm, 14.0 ± 0.0 mm, 22.0 ± 0.0 mm, and 26.0 ± 0.0 mm). Similarly, Sulfamethoxazole showed the maximum inhibition of *P. aeruginosa* and *S. epidermidis* (18.0 ± 0.0 mm, 18.0 ± 0.0 mm), while the lowest inhibition was recorded for other tested bacterial pathogens. Amoxicillin showed moderate inhibition of *P. aeruginosa, K. pneumoniae,* and *Staphylococcus epidermidis* (6.0 ± 0.0 mm, 8.0 ± 0.0 mm, and 9.0 ± 0.0 mm).

### 3.4. Synergistic Effect of Earthworm Extracts and Antibiotics

**Figure 4:**
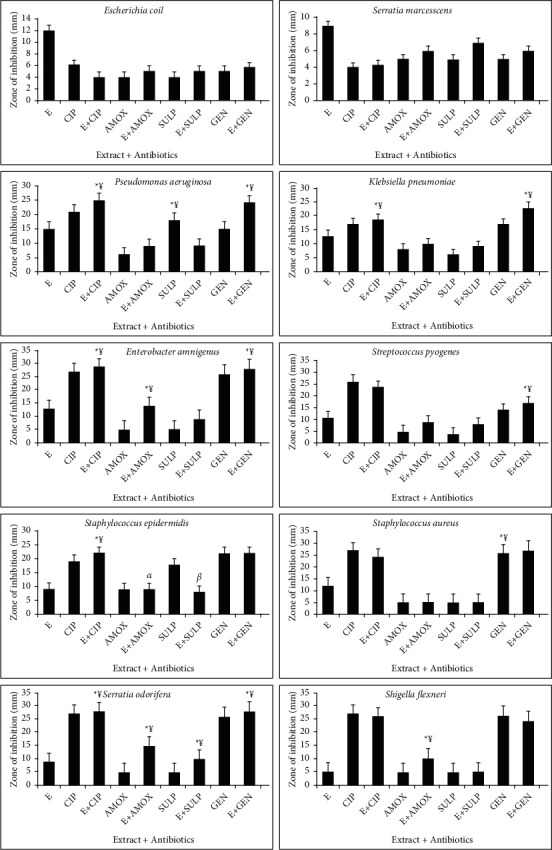
Interactions of *Amynthas corticis* extract along with antibiotics against bacterial pathogens. Synergistic effect (¥); additive effect (*a*); antagonistic effect (*β*)^*∗*^ at *p* ≤ 0.001.

**Figure 5:**
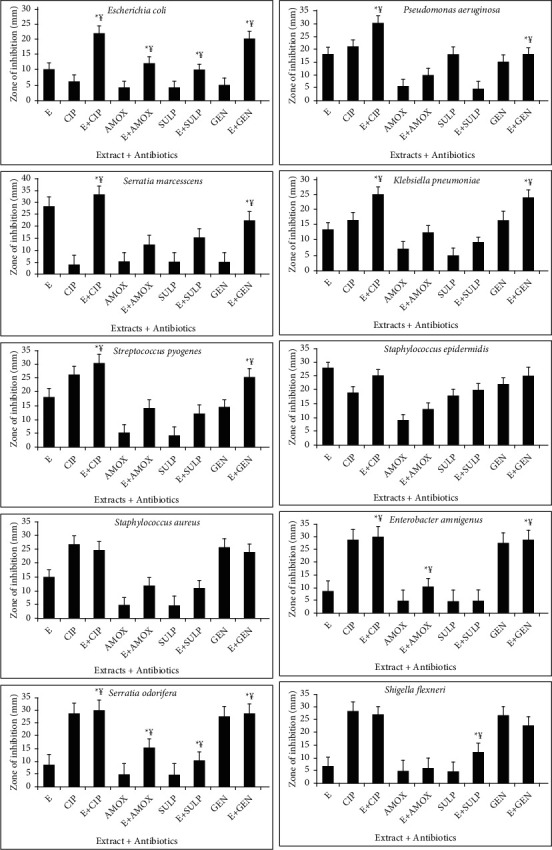
Interactions of extract of *Allolobophora chlorotica* along with antibiotics against bacterial pathogens. Synergistic effect (¥) was recorded (^*∗*^*p* ≤ 0.001).

**Figure 6:**
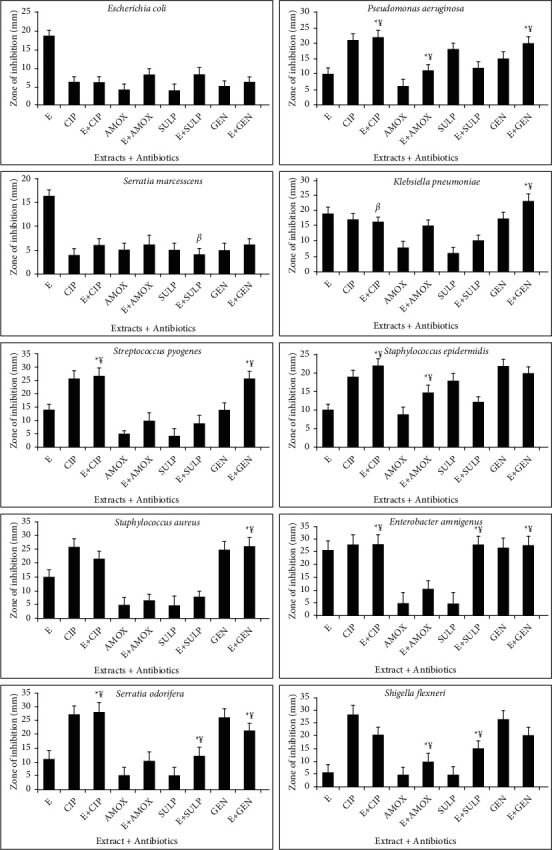
Interactions of extract of *Aporrectodea rosea* along with antibiotics against bacterial pathogens. Synergistic effect (¥) was recorded (^*∗*^*p* ≤ 0.001).

**Figure 7:**
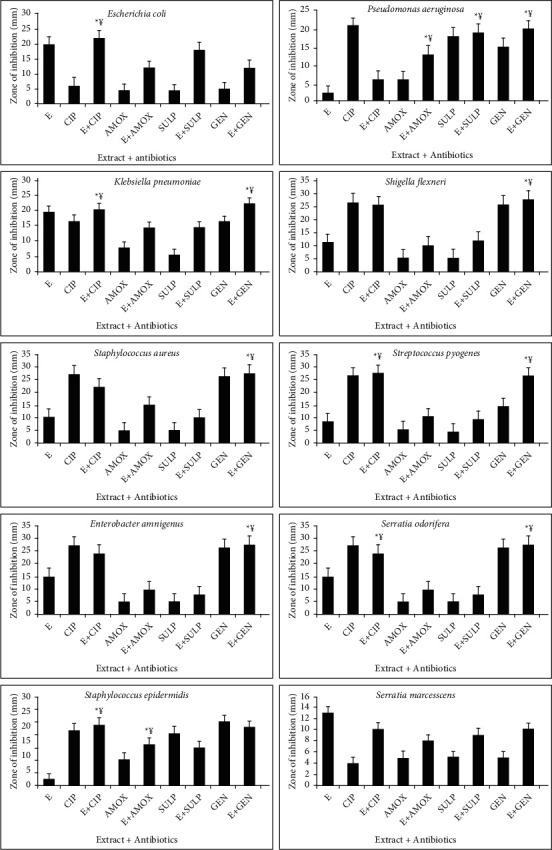
Interactions of *E. fetida* along with antibiotics against bacterial pathogens. Synergistic effect (¥) was recorded (^*∗*^*p* ≤ 0.001).

The combined effect of standard antibiotics and earthworm extract shows effective results. Three types of interactions such as synergistic, antagonist, and additive interactions are observed during the combination of extract and various antibiotics. Synergistic effect (¥) indicates the greater effects of two compounds when taken together than the sum of their separate effect. Additive effect (*α*) means the effect of two compounds is equal to the sum of the effect of two compounds taken separately. Antagonistic effect (*β*) shows the effect of two compounds is less than the sum of the effect of two compounds taken individually of each other (Figures 4–7). For the synergistic assay, only four earthworm species extracts such as *Amynthas corticis*, *Eisenia fetida, Aporrectodea rosea*, and *Allolobophora chlorotica* were selected, which showed maximum inhibition of bacterial pathogens. Similarly, out of 14 bacterial pathogens, only ten were selected for this assay.

### 3.5. Synergistic Effect of *Amynthas corticis* Extract and Antibiotics

Three types of interactions were recorded when *A. corticis* extract was applied with antibiotics (Figure 4). When the *A. corticis* extract was combined with Ciprofloxacin, they exhibited a significant (*p* ≤ 0.001) synergistic effect against *K. pneumoniae, P. aeruginosa,* and *S. epidermidis*. On the other hand, Ciprofloxacin showed antagonistic effect against *E. coli* when combined with extract. Moreover, Ciprofloxacin showed a significant (*p* ≤ 0.001) synergistic effect against *E. amnigenus*, *P. aeruginosa*, *S. marcescens*, *K. pneumoniae, S. odorifera,* and *S. pyogenes.* Similarly, the synergistic effect of gentamycin was also observed against *K. pneumoniae, E. amnigenus*, *S. pyogenes, S. aureus, S. odorifera*, and *P. aeruginosa* when combined with extract (at *p* ≤ 0.001). Amoxicillin along with extract showed an additive effect against *S. epidermidis*. On the other hand, the antagonistic effect of Sulfamethoxazole was recorded against *P. aeruginosa* and *S. epidermidis* (Figure 4). The significant (*p* ≤ 0.001) synergistic effect of amoxicillin was recorded against *E. amnigenus*, *S. odorifera,* and *S. flexneri.*

### 3.6. Synergistic Effect of *Allolobophora chlorotica* Extract and Antibiotics

Only synergistic effect was recorded against *E. coli* and *S. odorifera* when all antibiotics were applied with *A. chlorotica* extract at *p* ≤ 0.001 (Figure 5). Similarly, gentamycin showed the synergistic effect against *P. aeruginosa*, *K. pneumoniae, E. amnigenus*, *S. odorifera,* and *S. pyogenes.* Amoxicillin showed the synergistic effect against *E. amnigenus* and *S. odorifera.* On the other hand, no additive and antagonistic effect was recorded when antibiotics were applied with *A. chlorotica* extract (Figure 5).

### 3.7. Synergistic Effect of *Aporrectodea rosea* Extract and Antibiotics

The combination of *A. rosea* extract and standard antibiotics showed both synergistic and antagonistic effects (Figure 6). In the case of *P. aeruginosa* synergistic effect was recorded when Ciprofloxacin, amoxicillin, and gentamycin were applied with the extract at *p* ≤ 0.001. Similarly, synergistic effect against *K. pneumoniae, S. aureus, E. amnigenus*, *S. odorifera,* and *S. pyogenes* was recorded using gentamicin along with *A. rosea* extract. Additive effect was recoded against *S. marcescens* and *K. pneumoniae* when Sulfamethoxazole and Ciprofloxacin were used in combination. In the case of *S. epidermidis*, Ciprofloxacin and amoxicillin showed the synergistic effect at *p* ≤ 0.001. The synergistic effect of Ciprofloxacin and Sulfamethoxazole along with extract was also recorded against *E. amnigenus* (Figure 6).

### 3.8. Synergistic Effect of *Eisenia fetida* Extract and Antibiotics

The synergistic effect against *E. coli, K. pneumonia, S. epidermidis, S. odorifera,* and *S. pyogenes* was recorded when Ciprofloxacin was combined with *E. fetida* extract at *p* ≤ 0.001. Similarly, synergistic effect against *P. aeruginosa, K pneumonia, S. flexneri, S. aureus, S. pyogenes, S. odorifera,* and *E. amnigenus* was observed when gentamycin was applied with the extract (Figure 7). Amoxicillin showed a significant synergistic effect against *P. aeruginosa* and *S. epidermidis* at *p* ≤ 0.001. On the other hand, no additive and antagonistic effects were recorded when antibiotics were applied with *E. fetida* extract (Figure 7).

### 3.9. Cell Viability Assay

Interesting results were recorded when earthworm extracts were applied against tested bacterial pathogens. Results revealed that all earthworm extracts significantly inhibit the cell proliferation at the exponential phase of bacterial growth compared to both negative (only pathogen caring medium) and positive (Chloramphenicol) controls (Table 2). The values recorded at 570 nm for all the ten different earthworm species ranged within 0.0–0.5, values for negative control were recorded within 0.0–3.0, and values for positive control were measured within 0.0–1.5 (Table 2).

### 3.10. Biofilm Inhibition Effect

Biofilm inhibition results revealed that all the earthworm extracts reduced the biofilm formation compared to the control (nutrient broth having bacteria growth) and tested antibiotics (Table 2). *Amynthas corticis* extract significantly reduced the biofilm of *S. epidermidis, K. pneumonia, S. pyogenes, S. odorifera,* and *L. curvatus. Amynthas gracilis* extract reduced the biofilm of *S. marcescens, S. aureus,* and *S. pyogenes. Pheretima posthuma* extract reduced the biofilm of *E. coli, S. marcescens, K. pneumonia,* and *S. typhimurium. Eisenia fetida* extract inhibits the biofilm of *P. aeruginosa (a)* and *S. warneri. Aporrectodea rosea* showed the biofilm reduction of *E. coli, S. marcescens,* and *P. aeruginosa (b). Allolobophora chlorotica* indicated the reduction of biofilm of *K. pneumonia, P. aeruginosa (b),* and *S. warneri. Aporrectodea trapezoides* extract showed the biofilm reduction of *E. coli, S. marcescens, S. odorifera, S. typhimurium,* and *S. warneri. Polypheretima elongata* reduced the biofilm of *S. epidermidis* and *P. aeruginosa (b). Pheretima hawayana* reduced the *L. curvatus* biofilm only. *Aporrectodea caliginosa* showed the biofilm reduction of almost all tested bacterial pathogens.

### 3.11. Antioxidant Potential Effect

The antioxidant potential effect of all earthworm extracts was screened via DPPH and ABTS scavenging assays. *A. corticis, A. gracilis, A. rosea, A. chlorotica, P. elongata*, and *A. trapezoides* showed maximum DPPH scavenging potential effect. On the other hand, *P. elongata* indicated the same DPPH and ABTS scavenging potential. The highest value of antioxidant potential was recorded for *P. elongata* as 85% for both ABTS and DPPH scavenging assay. For *A. corticis* maximum scavenging potential was recorded as 80.74% and 77.5% for ABTS and DPPH, respectively. For *A. gracilis* 72% scavenging potential was recorded while 19% was recorded in the case of DPPH assay. *P. posthuma* indicated 81.57% potential through ABTS while indicating 19% potential through DPPH assay. *E. fetida* extracts showed 54.4% and 56% potential for ABTS and DPPH scavenging assay, respectively. In the case of *A. rosea*, 49.53% and 76% potential were measured through ABTS and DPPH scavenging assay. *A. chlorotica* indicated 10.45% and 70% values for ABTS and DPPH assay, respectively. *A. trapezoids* showed the lowest potential at 8.88% and 41% for ABTS and DPPH scavenging assay. *A. caliginosa* also showed minimum scavenging potential as 13.82% and 18% through ABTS and DPPH assay. For *P. hawayana* ABTS and DPPH scavenging potential were measured as 15.5% and 48%, respectively.

### 3.12. TLC Bioautography

The chemical constituents present in earthworm extracts were evaluated by thin-layer chromatography (TLC) by using precoated silica gel plates. To get the maximum efficient separation of components, five different solvent systems were used. Only the lipid biomolecules were indicated through TLC-developed plates in the form of movement of fat droplets in solvent system A, B, and C and not indicated in solvent system D and E. Bioautography was performed against *S. epidermidis*, *E. coli*, and *K. pneumonia* which showed good sensitivity towards *P. posthuma, E. fetida,* and *A. rosea* extracts. Bioautography revealed clear zones of bacterial growth inhibition on each TLC strip after treatment with TBTB with purple background indicating one or more bioactive antimicrobial compounds in earthworm extracts. It was observed that lipid biomolecules have a potent antibacterial effect against these tested bacterial pathogens.

### 3.13. Anticoagulant Activity

Results showed the mean coagulation time of plasma when treated with earthworm extracts. Negative control has a mean coagulation time of 2 min: 20 s. The findings showed that the coagulation time of plasma increases with the addition of earthworm extract. *P. hawayana* extracts prolonged the clot formation time more efficiently than all other species at 15 min : 18 sec and *A. corticis* also increases the prothrombin time more efficiently which was recorded as 12 min : 34 sec. *A. trapezoids* extracts also increased the clotting time as 11 min : 30 sec while *A. caliginosa* showed clotting time as 10 min : 41 sec. *P. posthuma* and *A. rosea* indicated a moderate increase in prothrombin time measured as 6 min : 18 sec and 6 min, respectively, while *E. fetida, P. elongata,* and *A. chlorotica* indicated low prothrombin time as 5 min : 50 sec, 4 min : 45 sec, and 4 min, respectively.

## 4. Discussion

The extract of earthworm species has been used for the treatment of various diseases including anti-inflammatory, antioxidant, antitumor, and antibacterial diseases, as a food ingredient (worm meal), in Traditional Chinese Medicine, and in Japan, Vietnam, and Korea [66, 67]. Earthworms are terrestrial, important soil-dwelling organisms and considered ecosystem engineers [68]. The results of the current study prove that extract of various earthworm species can inhibit bacterial growth and have potent antioxidant and anticoagulant effects, and our results are consistent with the outcomes of Bansal et al. [69] and Bansal et al. [70].

The antibacterial results revealed that the bactericidal effect of earthworm species varied against both Gram (+) and Gram (−) bacterial isolates. Even some earthworm extracts did not affect tested bacteria. This variation may be due to the type of bacterial isolates, cell wall composition of tested bacteria, concentration and quality of extracts used, nature and presence of the bioactive compound in the extract, and the type of extract used. But one thing that is interesting is that the antibacterial activity of earthworm species is attributed to the presence of bioactive substances existing in the earthworm extracts. Results revealed that different earthworm species showed the maximum inhibition of tested bacterial isolates; for example, *Eisenia fetida* extract showed the maximum inhibition of *E. coli*, *S. marcescens*, *K. pneumonia*, *S. aureus, E. amnigenus*, *S. odorifera*, and *S. flexneri, Polypheretima elongata* showed the maximum growth inhibition of *K. pneumoniae*, *E. amnigenus*, and *S. flexneri, Allolobophora chlorotica* indicated the maximum inhibition of *E. coli, S. marcescens*, *S. aureus, E. amnigenus*, and *S. odorifera, Aporrectodea rosea e*xtract showed maximum inhibition of *E. coli, S. marcescens, K. pneumoniae, S. pyogenes*, and *S. aureus, Pheretima posthuma* extract indicated the maximum growth inhibition of *E. coli, S. marcescens*, *K. pneumonia*, *S. aureus*, *S. pyogenes*, *S. odorifera*, and *S. flexneri,* and *Amynthas corticis* extract showed the maximum inhibition of *E. coli, S. marcescens*, and *E. amnigenus.* Our findings agree with the outcomes of previous literature that the coelomic fluid of earthworms contains bioactive compounds that participate in various biological activities [71–73].

Lumbricin-PG, the antimicrobial peptide, was identified from *Pheretima guillelmi* earthworm [74]. Bansal et al. [75] also demonstrated the antimicrobial activity of *Eudrilus eugeniae*. The outcomes of the current research are consistent with the findings of previous literature as Vasanthi et al. [76] presented the antimicrobial activity of *Eudrilus eugeniae* against *S. aureus;* Kathirewari et al. [47] found the antimicrobial effect of coelomic fluid of earthworm against microbes; Istiqumah et al. [48] studied the antibacterial activity of *Lumbricus rubellus* extracts against *E. coli, S. aureus, Salmonella pullorum,* and *P. aeruginosa*; Verma and Verma [49] found that coelomic fluid of earthworm *P. posthumous* had maximum antibacterial activity against *E. coli* (19.00 mm); Chauhan et al. [50] illustrated the antibacterial and antifungal activity of *Eudrilus eugeniae*; and Bhorgin and *Uma* [77] showed that ethanolic extract of earthworm powder possessed maximum antibacterial activity in comparison with petroleum ether and aqueous extract against *A. hydrophila*.

Hence, the current research sustained the findings of previous researchers that earthworm species possessed potential bioactive compounds, i.e., enzymes and antioxidants that play a key role in the growth inhibition of infectious pathogens. In the current research, it was observed that various earthworms possessed proteolytic, lipolytic, amylolytic, and antioxidant activities. Therefore, we can say that these bioactive substances (enzymes) and antioxidants may act as an antibacterial agent having various mechanisms/modes of actions (Figure 2), such as (1) the disruption/alteration/modification of plasma membrane and cell wall structure and function after attachment, (2) interruption of nucleic acid synthesis (DNA replication), (3) inhibition of RNA synthesis (transcription) and their functions, (4) interference with metabolic pathways, (5) inhibition of the protein synthesis and functions, and (6) generation of free radicals to disrupt cell membrane/cell wall, and anchoring to the cell membrane/cell wall [44, 78–80]. Our findings also agree with previous studies [81, 82]. They reported that a high concentration of free radicals damaged the proteins, lipids, and DNA. Antioxidants inhibit the potential digestive enzymes involved in the modulation of microbial composition [83, 84]. Various researchers demonstrated that cell envelope disruption is the primary target site because natural products can affect its integrity, fluidity, permeability, structure, and regulation of enzymes necessary for bacterial growth [85]. Similarly, Kim et al. [86] reported that oxidative stress can cause damage to the bacterial protein structure, intracellular system, and cell membrane against *E. coli* and *S. aureus.* Biofilm inhibition assay, cell proliferation inhibition assay, synergistic effect, and TLC-bio autography supported the results of agar well diffusion assay. Therefore, we can say that the development of new techniques, i.e., uses of enzymes with antibiotics, antimicrobial peptides production from vertebrates, invertebrates, and other microbes, and hybrid antibacterial drug production to prevent the emergence of bacterial pathogens are too effective, operative, and active [34, 37, 38]. Similarly, antioxidants production has also gained special attention over the last decades [87] due to the action as antimicrobial agents.

**Figure 8:**
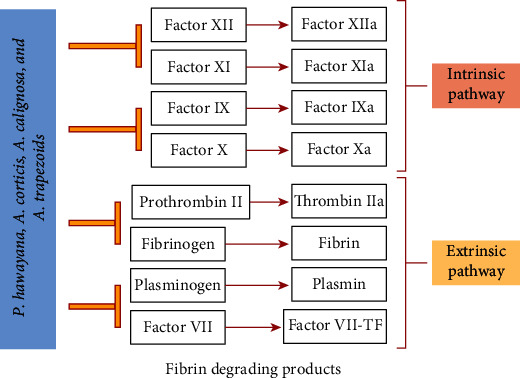
Role of earthworm extracts in inhibition of coagulation factors.

Blood circulation is essential for human survival [88]. During injury platelet aggregations, fibrinolysis, and blood coagulation processes are very important to restore the balance because any imbalance could lead to death or thromboembolic disorders [89]. Therefore, anticoagulants or anticoagulant therapy is crucial for the treatment and prevention of these disorders. Previous literature illustrated the isolation and characterization of anticoagulants from various earthworm species such as *Eisenia fetida, Pheretima posthumous, Lumbricus rubellus, Eudrilus eugeniae,* and *Pheretima guillemi* [89–93]. These anticoagulants are classified as thrombin inhibitors and FXa inhibitors. Thrombin is an important enzyme in the blood circulation system and plays a vital role in platelet activation, fibrinogen conversion to fibrin, and blood coagulation [89]. In the current research, earthworm extracts have been used for the screening of anticoagulant activity, and it was observed that *P. hawayana, A. corticis*, *A. caliginosa,* and *A. trapezoids* increase the prothrombin time more efficiently while *P. posthuma* and *A. rosea* indicated a moderate increase in prothrombin time. On the other hand, *E. fetida, P. elongata*, and *A. chlorotica* indicated low prothrombin time. So, we can say that the bioactive components of coelomic fluid of earthworm species may have interfered with both intrinsic and extrinsic pathways of coagulation; namely, they inhibit the activities of VII, II, IX, X, thrombin (thrombin IIa and prothrombin II), and Xa factors and played a key role in the fibrin degradation (Figure 8). Our outcomes agree with the previous literature that various anticoagulants such as lysenin, Lumbrokinase, and DPf3 (antithrombotic protein) can be isolated from earthworms and could be used in anticoagulant therapies [89–93].

## 5. Conclusion

We concluded that all earthworm species have antibacterial, antioxidant, and anticoagulant activities, and these findings can be used as a basis for the prevention of multidrug-resistant emergence, and production of animal-based anticoagulant agents. Furthermore, *in vivo* studies should be needed to explore the inhibition mechanisms of coagulation by using earthworm extracts.

## Figures and Tables

**Table 1:** Minimum inhibitory concentrations of earthworm extracts against bacterial pathogens via agar well diffusion method.

Extracts⟶ pathogens ↓	Zone of inhibition (*M* ± SD) in mm									
	*Amynthas corticis*	*Amynthas gracilis*	*Pheretima posthuma*	*Eisenia fetida*	*Aporrectodea rosea*	*Allolobophora chlorotica*	*Aporrectodea trapezoid*	*Polypheretima elongata*	*Aporrectodea caliginosa*	*Pheretima hawayana*
0.1 mg/ml concentration										
*E. coli*	7.0 ± 0.0^*∗∗*^	5.0 ± 0.0^*∗*^	12.0 ± 0.0^*∗∗∗*^	8.0 ± 0.0^*∗∗*^	5.0 ± 0.0^*∗*^	8.66 ± 0.47^*∗∗*^	1.66 ± 0.47^*∗∗*^	1.66 ± 0.77^*∗∗*^	2.0 ± 0.0^*∗*^	1.66 ± 0.77^*∗*^
*S. marcescens*	2.66 ± 0.47^*∗∗*^	2.0 ± 0.0^*∗*^	13.0 ± 0.0^*∗∗∗*^	8.0 ± 0.0^*∗∗*^	2.33 ± 0.47^*∗∗*^	9.33 ± 0.47^*∗∗*^	3.33 ± 0.47^*∗∗*^	3.33 ± 0.47^*∗∗*^	2.66 ± 0.47^*∗∗*^	3.33 ± 0.47^*∗*^
*K. pneumonia*	3.66 ± 0.47^*∗∗*^	2.66 ± 0.47^*∗∗*^	16.0 ± 0.0^*∗∗∗*^	14.0 ± 0.0^*∗∗∗*^	3.0 ± 0.0^*∗∗*^	6.0 ± 0.0^*∗∗*^	5.66 ± 0.47^*∗∗*^	7.33 ± 0.47^*∗∗*^	4.66 ± 0.47^*∗∗*^	2.33 ± 0.47^*∗*^
*S. aureus*	3.66 ± 0.47^*∗∗*^	0.0 ± 0.0	7.0 ± 0.0^*∗∗∗*^	9.33 ± 0.47^*∗∗*^	6.0 ± 0.0^*∗∗*^	9.33 ± 0.47^*∗∗*^	3.33 ± 0.47^*∗∗*^	3.33 ± 0.47^*∗∗*^	0.0 ± 0.0	4.66 ± 0.47^*∗*^
*S. pyogenes*	1.0 ± 0.0^*∗*^	3.0 ± 0.0^*∗*^	9.0 ± 0.0^*∗∗*^	2.0 ± 0.0^*∗*^	1.0 ± 0.0^*∗*^	4.66 ± 0.47^*∗∗*^	4.33 ± 0.47^*∗*^	2.33 ± 0.47^*∗*^	0.0 ± 0.0	2.66 ± 0.47^*∗*^
*P. aeruginosa*	0.0 ± 0.0	0.0 ± 0.0	0.0 ± 0.0	0.0 ± 0.0	0.0 ± 0.0	0.0 ± 0.0	0.0 ± 0.0	0.0 ± 0.0	0.0 ± 0.0	0.0 ± 0.0
*S. epidermidis*	0.0 ± 0.0	0.0 ± 0.0	0.0 ± 0.0	0.0 ± 0.0	0.0 ± 0.0	0.0 ± 0.0	0.0 ± 0.0	0.0 ± 0.0	0.0 ± 0.0	0.0 ± 0.0
*E. amnigenus*	7.66 ± 0.47^*∗∗*^	0.0 ± 0.0	12.0 ± 0.0^*∗∗∗*^	9.0 ± 0.0^*∗∗*^	2.33 ± 0.47^*∗*^	15.0 ± 0.0^*∗∗∗*^	8.66 ± 0.47^*∗∗*^	11.33 ± 0.47^*∗∗∗*^	3.66 ± 0.47^*∗*^	3.66 ± 0.47^*∗*^
*S. odorifera*	4.0 ± 0.0^*∗*^	3.0 ± 0.0^*∗∗*^	6.0 ± 0.0^*∗∗*^	9.66 ± 0.47^*∗∗*^	2.33 ± 0.47^*∗*^	8.66 ± 0.47^*∗∗*^	3.33 ± 0.47^*∗∗*^	2.0 ± 0.0^*∗*^	2.0 ± 0.0^*∗*^	2.66 ± 0.47^*∗*^
*S. typhimurium*	2.33 ± 0.47 ^*∗*^	2.0 ± 0.0^*∗*^	3.0 ± 0.0^*∗*^	2.0 ± 0.0^*∗*^	10.0 ± 0.0^*∗*^	3.0 ± 0.0^*∗∗*^	3.33 ± 0.47^*∗∗*^	1.33 ± 0.47^*∗*^	3.33 ± 0.47^*∗∗*^	7.0 ± 0.0^*∗∗*^
*S. flexneri*	2.0 ± 0.0^*∗*^	2.0 ± 0.0^*∗*^	9.0 ± 0.47^*∗∗*^	3.0 ± 0.0^*∗∗*^	3.66 ± 0.47^*∗*^	3.0 ± 0.0^*∗∗*^	4.66 ± 0.47^*∗∗*^	8.33 ± 0.47^*∗∗*^	2.33 ± 0.47^*∗∗*^	7.0 ± 0.0^*∗∗*^
*P. aeruginosa*	0.0 ± 0.0	0.0 ± 0.0	0.0 ± 0.0	0.0 ± 0.0	0.0 ± 0.0	0.0 ± 0.0	0.00 ± 0.0	0.0 ± 0.0	0.0 ± 0.0	0.0 ± 0.0
*S. warneri*	0.0 ± 0.0	0.0 ± 0.0	0.0 ± 0.0	0.0 ± 0.0	0.0 ± 0.0	0.0 ± 0.0	0.00 ± 0.0	0.0 ± 0.0	0.0 ± 0.0	0.0 ± 0.0
*L. curvatus*	0.0 ± 0.0	0.0 ± 0.0	0.0 ± 0.0	0.0 ± 0.0	0.0 ± 0.0	0.0 ± 0.0	0.00 ± 0.0	0.0 ± 0.0	0.0 ± 0.0	0.0 ± 0.0

0.5 mg/ml concentration										
*E. coli*	11.43 ± 0.41^*∗∗∗*^	5.0 ± 0.0^*∗*^	16.0 ± 0.0^*∗∗∗*^	15.0 ± 0.0^*∗∗∗*^	5.0 ± 0.0^*∗*^	14.0 ± 0.0^*∗∗∗*^	3.0 ± 0.0^*∗*^	2.5 ± 0.4^*∗*^	3.5 ± 0.4^*∗*^	4.16 ± 0.23^*∗*^
*S. marcescens*	4.33 ± 0.47^*∗*^	2.0 ± 0.0^*∗*^	17.0 ± 0.0^*∗∗∗*^	10.0 ± 0.0^*∗∗*^	3.0 ± 0.0^*∗*^	10.33 ± 0.47^*∗∗*^	4.33 ± 0.47^*∗*^	4.33 ± 0.47^*∗*^	3.0 ± 0.0^*∗*^	4.16 ± 0.23^*∗*^
*K. pneumonia*	4.33 ± 0.47^*∗*^	3.0 ± 0.0^*∗*^	18.0 ± 0.0^*∗∗∗*^	15.0 ± 0.0^*∗∗∗*^	4.33 ± 0.47^*∗*^	6.0 ± 0.0^*∗∗*^	5.66 ± 0.0.47^*∗∗*^	7.33 ± 0.47^*∗∗*^	5.66 ± 0.47^*∗∗*^	4.33 ± 0.47^*∗*^
*S. aureus*	3.0 ± 0.0^*∗*^	1.66 ± 0.47^*∗*^	9.0 ± 0.0^*∗∗*^	7.33 ± 0.47^*∗∗*^	6.0 ± 0.0^*∗∗*^	11.0 ± 0.0^*∗∗∗*^	4.33 ± 0.47^*∗*^	4.33 ± 0.47^*∗*^	0.0 ± 0.0	4.66 ± 0.47^*∗*^
*S. pyogenes*	3.0 ± 0.0^*∗*^	3.0 ± 0.0^*∗*^	11.0 ± 0.0^*∗∗∗*^	4.0 ± 0.0^*∗*^	2.0 ± 0.0^*∗*^	4.66 ± 0.47^*∗∗*^	4.33 ± 0.47^*∗*^	4.33 ± 0.47^*∗*^	2.0 ± 0.0^*∗*^	4.66 ± 0.47^*∗*^
*P. aeruginosa*	0.0 ± 0.0	0.0 ± 0.0	1.0 ± 0.0^*∗*^	1.0 ± 0.0^*∗*^	0.0 ± 0.0	0.0 ± 0.0	0.0 ± 0.0	0.0 ± 0.0	0.0 ± 0.0	0.0 ± 0.0
*S. epidermidis*	0.0 ± 0.0	0.0 ± 0.0	0.0 ± 0.0	0.0 ± 0.0	0.0 ± 0.0	0.0 ± 0.0	1.66 ± 0.47^*∗*^	0.0 ± 0.0	0.0 ± 0.0	0.0 ± 0.0
*E. amnigenus*	9.0 ± 0.0^*∗∗*^	0.0 ± 0.0	14.0 ± 0.0^*∗∗∗*^	10.0 ± 0.0^*∗∗*^	2.33 ± 0.47^*∗*^	16.0 ± 0.0^*∗∗∗*^	8.66 ± 0.47^*∗∗*^	12.0 ± 0.0.0^*∗∗∗*^	4.66 ± 0.47^*∗*^	4.66 ± 0.47^*∗*^
*S. odorifera*	5.0 ± 0.0^*∗*^	5.0 ± 0.0^*∗∗*^	7.0 ± 0.0^*∗∗*^	10.66 ± 0.47^*∗∗∗*^	2.33 ± 0.47^*∗*^	10.0 ± 0.0^*∗*^	3.33 ± 0.47^*∗∗*^	2.0 ± 0.0^*∗*^	2.0 ± 0.0^*∗*^	2.66 ± 0.47^*∗*^
*S. typhimurium*	2.33 ± 0.47 ^*∗*^	2.0 ± 0.0^*∗*^	3.0 ± 0.0^*∗*^	3.0 ± 0.0^*∗*^	11.0 ± 0.0^*∗∗*^	3.0 ± 0.0^*∗∗*^	3.33 ± 0.47^*∗∗*^	2.66 ± 0.47^*∗*^	4.33 ± 0.47^*∗∗*^	7.0 ± 0.0^*∗∗*^
*S. flexneri*	2.0 ± 0.0^*∗*^	2.0 ± 0.0^*∗*^	9.0 ± 0.47^*∗∗*^	3.0 ± 0.0^*∗∗*^	3.66 ± 0.47^*∗*^	3.0 ± 0.0^*∗∗*^	4.66 ± 0.47^*∗∗*^	8.33 ± 0.47^*∗∗*^	2.33 ± 0.47^*∗∗*^	7.0 ± 0.0^*∗∗*^
*P. aeruginosa*	0.0 ± 0.0	0.0 ± 0.0	0.0 ± 0.0	0.0 ± 0.0	0.0 ± 0.0	0.0 ± 0.0	0.00 ± 0.0	0.0 ± 0.0	0.0 ± 0.0	0.0 ± 0.0
*S. warneri*	0.0 ± 0.0	0.0 ± 0.0	0.0 ± 0.0	0.0 ± 0.0	0.0 ± 0.0	0.0 ± 0.0	0.00 ± 0.0	0.0 ± 0.0	0.0 ± 0.0	0.0 ± 0.0
*L. curvatus*	0.0 ± 0.0	0.0 ± 0.0	0.0 ± 0.0	0.0 ± 0.0	0.0 ± 0.0	0.0 ± 0.0	0.00 ± 0.0	0.0 ± 0.0	0.0 ± 0.0	0.0 ± 0.0

1.0 mg/ml concentration										
*E. coli*	14.66 ± 0.57^*∗∗∗*^	9.0 ± 1.0^*∗∗*^	25.0 ± 0.0^*∗∗∗*^	20.0 ± 0.0^*∗∗∗*^	8.0 ± 0.0^*∗∗*^	20.0 ± 0.0^*∗∗∗*^	2.33 ± 0.57^*∗*^	8.33 ± 0.57^*∗∗*^	9.33 ± 0.57^*∗∗*^	9.66 ± 0.57^*∗∗*^
*S. marcescens*	11.66 ± 0.57^*∗∗∗*^	5.0 ± 0.0^*∗*^	22.0 ± 0.0^*∗∗∗*^	13.0 ± 0.0^*∗∗∗*^	5.33 ± 0.57^*∗∗*^	16.33 ± 0.57^*∗∗∗*^	6.33 ± 0.57^*∗∗*^	6.33 ± 0.57^*∗∗*^	5.66 ± 0.57^*∗∗*^	7.33 ± 0.57^*∗∗*^
*K. pneumonia*	7.66 ± 0.57^*∗∗*^	5.66 ± 0.57^*∗∗*^	25.0 ± 0.0^*∗∗∗*^	20.0 ± 0.0^*∗∗∗*^	7.0 ± 0.0^*∗∗*^	10.0 ± 0.0^*∗∗*^	8.33 ± 0.57^*∗∗*^	10.33 ± 0.57^*∗∗∗*^	9.66 ± 0.57^*∗∗*^	8.33 ± 0.57^*∗∗*^
*S. aureus*	6.0 ± 0.57^*∗∗*^	4.66 ± 0.57^*∗*^	15.0 ± 0.0^*∗∗∗*^	10.33 ± 0.57^*∗∗∗*^	10.0 ± 0.0^*∗∗*^	16.33 ± 0.57^*∗∗∗*^	6.33 ± 0.57^*∗∗*^	7.33 ± 0.57^*∗∗*^	2.0 ± 0.0^*∗*^	7.33 ± 0.57^*∗∗*^
*S. pyogenes*	7.0 ± 0.0^*∗∗*^	5.0 ± 0.0^*∗*^	15.0 ± 0.0^*∗∗∗*^	8.0 ± 0.0^*∗∗*^	5.0 ± 0.0^*∗*^	8.66 ± 0.57^*∗∗*^	6.33 ± 0.57^*∗∗*^	6.66 ± 0.57^*∗∗*^	5.0 ± 0.0^*∗*^	7.33 ± 0.57^*∗∗*^
*P. aeruginosa*	2.0 ± 0.0^*∗*^	2.0 ± 0.0^*∗*^	2.33 ± 0.0^*∗*^	2.33 ± 0.57^*∗*^	2.0 ± 0.0^*∗*^	2.0 ± 0.0^*∗*^	2.00 ± 0.0^*∗*^	2.0 ± 0.0^*∗*^	2.0 ± 0.0^*∗*^	2.0 ± 0.0^*∗*^
*S. epidermidis*	2.33 ± 0.57^*∗*^	2.0 ± 0.0^*∗*^	2.0 ± 0.0^*∗*^	2.0 ± 0.0^*∗*^	2.0 ± 0.0^*∗*^	2.0 ± 0.0^*∗*^	2.33 ± 0.57^*∗*^	2.0 ± 0.0^*∗*^	2.0 ± 0.0^*∗*^	2.0 ± 0.0^*∗*^
*E. amnigenus*	12.66 ± 0.57^*∗∗∗*^	2.0 ± 0.0^*∗*^	25.0 ± 0.0^*∗∗∗*^	15.0 ± 0.0^*∗∗∗*^	8.33 ± 0.57^*∗∗*^	25.0 ± 0.0^*∗∗∗*^	10.66 ± 0.57^*∗∗*^	13.33 ± 0.57^*∗∗∗*^	7.66 ± 0.57^*∗∗*^	9.66 ± 0.57^*∗∗*^
*S. odorifera*	9.0 ± 0.0^*∗*^	7.0 ± 1.73^*∗∗*^	10.0 ± 0.0^*∗∗*^	12.66 ± 0.57^*∗∗∗*^	8.33 ± 0.57^*∗∗*^	10.66 ± 0.57^*∗∗*^	5.33 ± 0.57^*∗∗*^	5.0 ± 0.0^*∗*^	5.0 ± 0.0^*∗*^	7.66 ± 0.57^*∗∗*^
*S. typhimurium*	6.33 ± 1.15^*∗*^	4.0 ± 0.0^*∗*^	7.0 ± 0.0^*∗∗*^	6.0 ± 0.0^*∗∗*^	16.0 ± 0.0^*∗∗∗*^	6.0 ± 0.0^*∗∗*^	6.33 ± 0.57^*∗∗*^	3.33 ± 0.57^*∗*^	9.33 ± 0.57^*∗∗*^	10.0 ± 0.0^*∗∗*^
*S. flexneri*	5.0 ± 0.0^*∗*^	5.0 ± 0.0^*∗*^	20.0 ± 0.57^*∗∗∗*^	11.33 ± 0.57^*∗∗∗*^	6.66 ± 0.57^*∗∗*^	6.0 ± 0.0^*∗∗*^	8.66 ± 0.57^*∗∗*^	10.33 ± 0.57^*∗∗∗*^	6.33 ± 0.57^*∗∗*^	10.0 ± 0.0^*∗∗*^
*P. aeruginosa*	2.0 ± 0.0^*∗*^	2.0 ± 0.0^*∗*^	2.0 ± 0.0^*∗*^	2.0 ± 0.0^*∗*^	2.0 ± 0.0^*∗*^	2.0 ± 0.0^*∗*^	2.00 ± 0.0^*∗*^	2.0 ± 0.0^*∗*^	2.0 ± 0.0^*∗*^	2.0 ± 0.0^*∗*^
*S. warneri*	2.0 ± 0.0^*∗*^	2.0 ± 0.0^*∗*^	2.0 ± 0.0^*∗*^	2.0 ± 0.0^*∗*^	2.0 ± 0.0^*∗*^	2.0 ± 0.0^*∗*^	2.00 ± 0.0^*∗*^	2.0 ± 0.0^*∗*^	2.0 ± 0.0^*∗*^	2.0 ± 0.0^*∗*^
*L. curvatus*	2.0 ± 0.0^*∗*^	2.0 ± 0.0^*∗*^	2.0 ± 0.0^*∗*^	2.0 ± 0.0^*∗*^	2.0 ± 0.0^*∗*^	2.0 ± 0.0^*∗*^	2.00 ± 0.0^*∗*^	2.0 ± 0.0^*∗*^	2.0 ± 0.0^*∗*^	2.0 ± 0.0^*∗*^
					Allolobophora chlorotica		Polypheretima elongata	Aporrectodea caliginosa	Pheretima hawayana	

Zone of inhibition expressed as “0” for no sensitivity, “^*∗*^” for low sensitivity, “^*∗∗*^” for moderate sensitivity, and “^*∗∗∗*^” for highest sensitivity.

**Table 2:** Biofilm inhibition and cell proliferation inhibition effects of earthworm species extracts against bacterial pathogens.

Bacterial pathogens↓ extracts ⟶	Biofilm inhibition effect											
	Absorbance at 550 nm (mean values)											
	Control	*A. corticis*	*A. gracilis*	*P. posthuma*	*E. fetida*	*A. rosea*	*A. chlorotica*	*A. trapezoid*	*P. elongata*	*A. caliginosa*	*P. hawayana*	Chloramphenicol
*Escherichia coli*	4.11	1.692	2.77	1.157	2.177	1.05	1.55	1.19	2.17	1.635	1.81	0.591
*Staphylococcus aureus*	3.62	1.791	0.51	1.616	1.77	2.89	1.98	4.01	1.87	1.456	3.31	0.531
*Staphylococcus epidermidis*	3.54	1.011	1.081	2.915	1.85	1.5	1.38	1.51	1.05	1.734	3.67	0.24
*Pseudomonas aeruginosa*	3.65	1.791	1.29	2.101	1.21	1.72	1.69	1.92	1.9	1.456	4.01	0.609
*Serratia marcescens*	3.52	1.989	0.518	1.306	2.915	1.05	1.21	1.23	1.87	1.325	3.92	0.526
*Klebsiella pneumonia*	4.02	0.875	1.434	1.291	1.876	1.58	1.03	1.67	1.58	1.542	3.77	0.513
*Streptococcus pyogenes*	3.91	1.05	0.491	2.35	2.78	2.06	1.65	1.68	3.77	1.563	3.5	0.349
*Enterobacter amnigenus*	3.62	1.919	2.242	2.912	2.38	1.59	2.91	2.81	1.66	1.392	2.52	0.66
*Serratia odorifera*	2.91	0.232	2.913	2.451	2.9	1.81	2.76	1.06	1.67	1.311	2.19	0.76
*Salmonella typhimurium*	2.37	1.92	2.275	1.396	2.31	1.9	2.96	1.09	2.81	1.55	1.71	0.56
*Shigella flexneri*	3.52	2.019	2.219	1.9	2.95	1.71	2.18	2.91	2.8	1.87	1.92	0.43
*Pseudomonas aeruginosa*	3.81	2.18	2.11	2.35	2.77	1.01	1.2	1.87	1.01	1.9	3.21	0.98
*Staphylococcus warneri*	3.71	2.91	1.375	2.801	1.06	2.18	1.01	1.14	1.85	1.82	2.93	0.77

Bacterial pathogens↓ extracts ⟶	Cell proliferation inhibition effect											
	Absorbance at 550 nm (mean values)											
*Escherichia coli*	1.692	0.255	0.275	0.191	0.376	0.223	0.313	0.283	0.274	0.301	0.206	0.71
*Staphylococcus aureus*	1.791	0.219	0.193	0.265	0.314	0.466	0.351	0.294	0.216	0.374	0.274	0.626
*Staphylococcus epidermidis*	1.011	0.269	0.29	0.194	0.393	0.275	0.275	0.254	0.284	0.298	0.175	0.232
*Pseudomonas aeruginosa*	1.791	0.129	0.193	0.293	0.336	0.483	0.355	0.281	0.315	0.374	0.274	1.017
*Serratia marcescens*	1.989	0.29	0.283	0.294	0.327	0.382	0.313	0.314	0.216	0.301	0.266	0.311
*Klebsiella pneumonia*	0.875	0.293	0.268	0.384	0.32	0.474	0.283	0.293	0.333	0.315	0.282	1.133
*Streptococcus pyogenes*	1.05	0.211	0.291	0.284	0.393	0.287	0.283	0.272	0.215	0.177	0.241	1.255
*Enterobacter amnigenus*	1.919	0.427	0.194	0.188	0.371	0.327	0.261	0.294	0.196	0.164	0.171	0.278
*Serratia odorifera*	0.675	0.182	0.185	0.177	0.299	0.372	0.215	0.284	0.175	0.301	0.195	0.267
*Salmonella typhimurium*	1.92	0.277	0.286	0.213	0.295	0.326	0.283	0.236	0.214	0.292	0.207	0.307
*Shigella flexneri*	2.019	0.287	0.276	0.282	0.274	0.383	0.216	0.183	0.205	0.285	0.298	0.331
*Pseudomonas aeruginosa*	2.18	0.164	0.211	0.284	0.183	0.292	0.154	0.296	0.178	0.182	0.181	0.267
*Staphylococcus warneri*	2.91	0.274	0.193	0.287	0.194	0.165	0.218	0.273	0.204	0.174	0.154	0.323

## Data Availability

Data will be available on request.

## Additional Points

All experiments have been designed to avoid distress, unnecessary pain, and suffering to the experimental animals. All procedures were conducted following international regulations referred to as Wet op de dierproeven (Article 9) of Dutch Law.

## References

[1] Cotar A. I. (2013). An introduction to my research interests. *Clinical Microbiology*.

[2] Giangaspero M., Orusa R., Savini G. (2013). Serological survey to determine the occurrence of blue tongue virus, bovine leukemia virus and herpesvirus infections in the Japanese small ruminant population from northern districts. *Clinical Microbiology*.

[3] Sahil D., Otag F. (2013). Filamentous fungi isolated from clinical samples stored for a long time in the sand. *Clinical Microbiology Open Access*.

[4] Chlebicz A., Slizewska K. (2018). Campylobacteriosis, salmonellosis, yersiniosis, and listeriosis as zoonotic foodborne diseases: a review. *International Journal of Environmental Research and Public Health*.

[5] Illnait-Zaragozi M. T., Martínez R. E. V., Ferrer J. I. (2014). In vitro antifungal activity of crude hydro- alcoholic extract of petiveria alliacea L on clinical Candida isolates. *Clinical Microbiology*.

[6] Lovayová V., Vargová L., Habalová V. (2014). New Delhi metallo-beta-lactamase Ndm-1 producing *Klebsiella pneumoniae* in Slovakia. *Clinical Microbiology*.

[7] Bantawa K., Rai K., Subba Limbu D., Khanal H. (2018). Food-borne bacterial pathogens in marketed raw meat of dharan, eastern Nepal. *BMC Research Notes*.

[8] Assefa A., Bihon A. (2018). A systematic review and meta-analysis of prevalence of *Escherichia coli* in foods of animal origin in Ethiopia. *Heliyon*.

[9] Elmonir W., Abo-Remela M. E., Sobeih A. (2018). Public health risks of *Escherichia coli* and *Staphylococcus aureus* in raw bovine milk sold in informal markets in Egypt. *Journal of Infections in Developing*.

[10] Hemalata V. B., Virupakshaiah D. B. M. (2016). Isolation and identification of food borne pathogens from spoiled food samples. *International Journal of Current Microbiology and Applied Sciences*.

[11] Muthupandian S., Balajee R., Barabadi H. (2017). The prevalence and drug resistance pattern of extended spectrum 𝛽–lactamases (ESBLs) producing enterobacteriaceae in Africa. *Microbial Pathogenesis*.

[12] Otto M. (2009). Staphylococcus epidermidisthe accidental pathogen. *International Journal of Antimicrobl Agents*.

[13] Saravanan M., Niguse S., Abdulkader M. (2018). Review on emergence of drug-resistant tuberculosis (MDR & XDR-TB) and its molecular diagnosis in Ethiopia. *Microbial Pathogenesis*.

[14] Bobbarala V., Vadlapudi V. (2009). Abrus-precatorius, Seed extracts antimicrobial properties against clinically important bacteria. *International Journal of Pharmaceutical and Technology Research*.

[15] Golkar Z., Bagasra O., Pace D. G. (2014). Bacteriophage therapy: a potential solution for the antibiotic resistance crisis. *Journal of infection in developing countries*.

[16] Spellberg B., Gilbert D. N. (2014). The future of antibiotics and resistance: a tribute to a career of leadership by John Bartlett. *Clinical Infectious Diseases*.

[17] Michael C. A., Dominey-Howes D., Labbate M. (2014). The antimicrobial resistance crisis: causes, consequences, and management. *Frontiers in Public Health*.

[18] Gould I. M., Bal A. M. (2013). New antibiotic agents in the pipeline and how they can help overcome microbial resistance. *Virulence*.

[19] Read A. F., Woods R. J. (2014). Antibiotic resistance management. *Evolution, Medicine, and Public Health*.

[20] Mendes R. E., Deshpande L. M., Jones R. N. (2014). Linezolid update: stable in vitro activity following more than a decade of clinical use and summary of associated resistance mechanisms. *Drug Resistance Updates : Reviews and Commentaries in Antimicrobial and Anticancer Chemotherapy*.

[21] Miller W. R., Munita J. M., Arias C. A. (2014). Mechanisms of antibiotic resistance in enterococci. *Expert Review of Anti-infective Therapy*.

[22] Hollenbeck B. L., Rice L. B. (2012). Intrinsic and acquired resistance mechanisms in enterococcus. *Virulence*.

[23] Ramirez M. S., Tolmasky M. E. (2010). Aminoglycoside modifying enzymes. *Drug Resistance Updates*.

[24] Wilson D. N. (2014). Ribosome-targeting antibiotics and mechanisms of bacterial resistance. *Nature Reviews Microbiology*.

[25] Nikaido H. (2003). Molecular basis of bacterial outer membrane permeability revisited. *Microbiology and Molecular Biology Reviews*.

[26] Pagès J. M., James C. E., Winterhalter M. (2008). The porin and the permeating antibiotic: a selective diffusion barrier in Gram-negative bacteria. *Nature Reviews. Microbiology*.

[27] Roberts M. C. (2005). Update on acquired tetracycline resistance genes. *FEMS Microbiology Letters*.

[28] Rodríguez-Martínez J. M., Cano M. E., Velasco C. (2011). Plasmid-mediated quinolone resistance: an update. *Journal of Infection and Chemotherapy*.

[29] Poole K. (2005). Efflux-mediated antimicrobial resistance. *Journal of Antimicrobial Chemotherapy*.

[30] Katz L., Ashley G. W. (2005). Translation and protein synthesis: macrolides. *Chemical Reviews*.

[31] Bush K. (2013). Proliferation and significance of clinically relevant *β*-lactamases. *Annals of the New York Academy of Sciences*.

[32] D’Costa V. M., King C. E., Kalan L. (2011). Antibiotic resistance is ancient. *Nature*.

[33] Wilkinson A., Holmes S., Pitts K. SASP: A novel antibacterial DNA binding protein and its targeted delivery to Staphylococcus aureus.

[34] Tarkkanen A.-M., Heinonen T., Jogi R. (2009). P1A recombinant *β*-lactamase prevents emergence of antimicrobial resistance in gut microflora of healthy subjects during intravenous administration of ampicillin. *Antimicrobial Agents and Chemotherapy*.

[35] Randhawa G., Kullar J., Rajkumar J. (2011). Bioenhancers from mother nature and their applicability in modern medicine. *International Journal of Applied and Basic Medical Research*.

[36] Ejim L., Farha M. A., Falconer S. B. (2011). Combinations of antibiotics and nonantibiotic drugs enhance antimicrobial efficacy. *Nature Chemical Biology*.

[37] Coutinho H. D., Lôbo K. M., Bezerra D. A., Lôbo I (2008). Peptides and proteins with antimicrobial activity. *Indian Journal of Pharmacology*.

[38] Asahina Y., Nagae O., Sato T. AM-3005: Synthesis and in vitro antibacterial activity of novel mutilin-quinolone hybrid antibacterial agent (F1-2030).

[39] Meers P., Neville M., Malinin V. (2008). Biofilm penetration, triggered release and in vivo activity of inhaled liposomal amikacin in chronic *Pseudomonas aeruginosa* lung infections. *Journal of Antimicrobial Chemotherapy*.

[40] Ejaz K., Sadia H., Zia G. (2018). Biofilm reduction, cell proliferation, anthelmintic and cytotoxicity effect of green synthesised silver nanoparticle using Artemisia vulgaris extract. *IET Nanobiotechnology*.

[41] Zia G., Sadia H., Nazir S. (2018). In vitro studies on cytotoxic, DNA protecting, antibiofilm and antibacterial effects of biogenic silver nanoparticles prepared with bergenia ciliata rhizome extract. *Current Pharmaceutical Biotechnology*.

[42] Senhaji S., Lamchouri F., Toufik H. (2020). Phytochemical content, antibacterial and antioxidant potential of endemic plant anabasis aretioïdes coss. & Moq. (Chenopodiaceae). *BioMedical Research International*.

[43] Berdowska I., Zieliński B., Fecka I., Kulbacka J., Saczko J., Gamian A. (2013). Cytotoxic impact of phenolics from Lamiaceae species on human breast cancer cells. *Food Chemistry*.

[44] Papuc C., Goran G. V., Predescu C. N., Nicorescu V., Stefan G. (2017). Plant polyphenols as antioxidant and antibacterial agents for shelf-life extension of meat and meat products: classification, structures, sources, and action mechanisms. *Comprehensive Reviews in Food Science and Food Safety*.

[45] Gyawali R., Ibrahim S. A. (2014). Natural products as antimicrobial agents. *Food Control*.

[46] Cooper E. L., Ru B., Weng N. (2004). Earthworms: sources of antimicrobial and anticancer molecules. *Advances in Experimental Medicine & Biology*.

[47] Kathireswari P., Alakesan A., Abirami P. (2014). Antimicrobial activity of earthworm coelomic fluid against diseases causing microorganisms. *International Journal of Current Microbiology and Applied Sciences*.

[48] Istiqomah L., Herdian H., Damayanti E., Hayati S. N., Julendra H. (2012). Inhibitory of encapsulated earthworm extract (Lumbricus rubellus) on pathogenic bacteria in vitro. *Media Peternakan*.

[49] Verma Y. K., Verma M. K. (2012). Earthworm-a potential source for stable and potent antimicrobial compounds-isolation and purification study. *International Journal of Pharmacological Sciences*.

[50] Chauhan P. S., Tomar J., Prasad G. B. (2014). Evaluation of antimicrobial activity of earthworm Eudrilus eugeniae tissue extract. *Journal of Chemical and Pharmaceutical Research*.

[51] Edwards C. A. (2004). *Earthworm Ecology*.

[52] Andleeb S., Ejaz M., Awan U. A. (2016). In vitro screening of mucus and solvent extracts of Eisenia foetida against human bacterial and fungal pathogens. *Pakistan journal of pharmaceutical sciences*.

[53] Siddique U.-K., Shahzad N. (2013). In vitro screening of Medicinal Herbal extracts and Antibiotics against Bacteria isolated from Fish products at retail outlets. *British Microbiology Research Journal*.

[54] Awan U. A., Andleeb S., Kiyani A. (2013). Antimicrobial screening of traditional herbal plants and standard antibiotics against some human bacterial pathogens. *Pakistan journal of pharmaceutical sciences*.

[55] Rios J. L., Recio M. C., Villar A. (1988). Screening methods for natural products with antimicrobial activity: a review of the literature. *Journal of Ethnopharmacology*.

[56] Seeley H. W., Vandemark P. J., Lee J. J. (2001). *Microbes in Action: A Laboratory Manual of Microbiology*.

[57] Hammer K. A., Carson C. F., Riley T. V. (1999). Antimicrobial activity of essential oils and other plant extracts. *Journal of Applied Microbiology*.

[58] Bauer A. W., Kirby W. M. M., Sherris J. C., Turck M. (1966). Antibiotic susceptibility testing by a standardized single disk method. *American Journal of Clinical Pathology*.

[59] Prescott M. L., Harley J., Donald P. (1999). In Antimicrobial chemotherapy. *Microbiology*.

[60] O’Toole G. A. (2011). Microtiter dish biofilm formation assay. *Journal of Visualized Experiments*.

[61] Gerlier D., Thomasset N. (1986). Use of MTT colorimetric assay to measure cell activation. *Journal of Immunological Methods*.

[62] Rubens F. V. D. S., Wagner F. D. G. (2004). Antioxidant properties of complexes of flavonoids with metal ions. *Redox Report*.

[63] Re R., Pellegrini N., Proteggente A., Pannala A., Yang M., Rice-Evans C. (1999). Antioxidant activity applying an improved ABTS radical cation decolorization assay. *Free Radical Biology and Medicine*.

[64] Roopalatha U. C., Vijay-mala N. (2013). The phytochemical screening of the pericarp of fruits of Terminalia chebula Retz. *International Journal of Pharmacology and Biological Science*.

[65] Dandjesso C., Klotoa J. R., Doµgnon T. V. (2012). Phytochemistry and hemostatic properties of some medicinal plants sold as anti-hemorrhagic in Cotonou markets (Benin). *Indian Journal of Science and Technology*.

[66] Aldarraji M., Halimoon N., Majid N. M. (2013). Antioxidant activity and total phenolic content of earthworm paste of Lumbricus rubellus (red worm) and Eudrilus eugenia (African night crawler). *Journal of Entomology and Nematology*.

[67] Cooper E. L., Hirabayashi K. (2013). Origin of innate immune responses: revelation of food and medicinal applications. *Journal of traditional and Complementary Medicine*.

[68] Blouin M., Hodson M. E., Delgado E. A. (2013). A review of earthworm impact on soil function and ecosystem services. *European Journal of Soil Science*.

[69] Bansal N., Gupta R. K., Shashank K. (2016). Antimicrobial activity of earthworm extract, Eudrilus eugeniae against fish bacterial pathogens. *The Ecoscan*.

[70] Bansal N., Gupta R. K., Singh D., Shashank S. (2015). Comparative study of antibacterial activity of two different earthworm species, Perionyx excavatus and Pheretima posthuma against pathogenic bacteria. *Journal of Applied and Natural Science*.

[71] Cooper E. L., Balamurugan M., Huang C. Y. (2012). Earthworms dilong: ancient, inexpensive, noncontroversial models my help clarify approaches to integrated medicine emphasizing neuroimmuno systems. *Evidence-based Complementary and Alternative Medicine*.

[72] Mataušić-Pišl M., Tomičić M., Micek V. (2011). Influence of earthworm extract G-90 on the haemostasis in Wistar rats. *European Review for Medical and Pharmacological Sciences*.

[73] El-Din H., Omar M., Ibraheim Z. (2012). Anti-inflammatory, antipyretic and antioxidant activities of the earthworms extract. *Journal of Biology and Earth Science*.

[74] Li W., Li S., Zhong J., Zhu Z., Liu J., Wang W. (2011). A novel antimicrobial peptide from skin secretions of the earthworm, Pheretima guillelmi (Michaelsen). *Peptides*.

[75] Bansal N., Gupta R. K., Nehra V. (2018). Antibacterial effects of *Eisenia fetida*, earthworm extract against pathogenic bacteria isolated from *Cyprinus carpio*. *International Journal of Current Microbiology and Applied Sciences*.

[76] Vasanthi K., Chairman K., Ranjit-Singh A. J. A. (2013). Antimicrobial activity of earthworm (Eudrilus eugeniae) paste. *African Journal of Environmental Science and Technology*.

[77] Bhorgin A. J., Uma K. (2014). Antimicrobial activity of earthworm powder (lampito mauritii). *International Journal of Current Microbiology and Applied Sciences*.

[78] Mickymaray S., Alfaiz F. A., Paramasivam A. (2020). Efficacy and mechanisms of flavonoids against the emerging opportunistic nontuberculous Mycobacteria. *Antibiotics*.

[79] Farhadi F., Khameneh B., Iranshahi M., Iranshahy M. (2019). Antibacterial activity of flavonoids and their structure-activity relationship: an update review. *Phytotherapy Research*.

[80] Coppo E., Marchese A. (2014). Antibacterial activity of polyphenols. *Current Pharmaceutical Biotechnology*.

[81] Phaniendra A., Jestadi D. B., Periyasamy L. (2015). Free radicals: properties, sources, targets, and their implication in various diseases. *Indian Journal of Clinical Biochemistry*.

[82] Schieber M., Chandel N. S. (2014). ROS function in redox signaling and oxidative stress. *Current Biology*.

[83] Sun L., Wang Y., Miao M. (2020). Inhibition of *α*-amylase by polyphenolic compounds: substrate digestion, binding interactions and nutritional intervention. *Trends in Food Science & Technology*.

[84] Martinez-Gonzalez A. I., Díaz-Sánchez Á. G., Rosa L. A. d. l., Vargas-Requena C. L., Bustos-Jaimes I., Alvarez-Parrilla A. E. (2017). Polyphenolic compounds and digestive enzymes: in vitro non-covalent interactions. *Molecules*.

[85] Nazzaro F., Fratianni F., Coppola R. (2013). Quorum sensing and phytochemicals. *International Journal of Molecular Sciences*.

[86] Kim S. H., Lee H. S., Ryu D. S. (2011). Antibacterial activity of silver-nanoparticles against *Staphylococcus aureus* and *Escherichia coli*. *Korean Journal of Microbiology and Biotechnology*.

[87] Shahidi F., Ambigaipalan P. (2015). Phenolics and polyphenolics in foods, beverages and spices: antioxidant activity and health effects-a review. *Journal of Functional Foods*.

[88] Koh C. Y., Kini R. M. (2012). From snake venom toxins to therapeutics - cardiovascular examples. *Toxicon*.

[89] Fu Z., Zhang L., Liu X. (2013). Comparative proteomic analysis of the sun- and freeze-dried earthworm *Eisenia fetida* with differentially thrombolytic activities. *Journal of Proteomics*.

[90] Verma M. K., Pulicherla K. K. (2017). Broad substrate affinity and catalytic diversity of fibrinolytic enzyme from Pheretima posthumous-Purification and molecular characterization study. *International Journal of Biological Macromolecules*.

[91] Matausic-Pisl M., Tomičić M., Micek V. (2011). Influences of earthworm extract G-90 on haematological and haemostatic parameters in Wistar rats. *European Review for Medical and Pharmacological Sciences*.

[92] Wu Y., Ma Y., Hu S. (2019). Transcriptomic-proteomics-anticoagulant bioactivity integrated study of Pheretima guillemi. *Journal of Ethnopharmacology*.

[93] Murugan S., Umamaheswari S. (2021). Identification of lysenin protein function in coelomic fluid of eudrilus eugeniae. *European Journal of Molecular & Clinical Medicine*.

